# Novel approach for tracheal resection in Morquio a syndrome with end-stage critical airway obstruction: a UK case series

**DOI:** 10.1186/s13023-024-03253-3

**Published:** 2024-07-22

**Authors:** Johnny Kenth, Elizabeth Maughan, Colin R Butler, Jasleen Gabrie, Maral Rouhani, Benjamin Silver, Olumide K Ogunbiyi, Stuart Wilkinson, Reema Nandi, Robert Walker, Nagarajan Muthialu, Simon Jones, Richard Hewitt, Iain A Bruce

**Affiliations:** 1 Department of Paediatric Anaesthesia, Royal Manchester Children’s Hospital, Manchester University NHS Foundation Trust, Manchester, UK; 2 Divisions of Infection, Immunity and Respiratory Medicine, Faculty of Biology, Medicine and Health, University of Manchester, Manchester, UK; 3 National Paediatric Tracheal Service, Great Ormond Street Hospital for Children NHS Foundation Trust, London, UK; 4 UCL Great Ormond Street Institute of Child Health, UCL Great Ormond Street Institute of Child Health, Great Ormond Street Hospital for Children, UK; 5 Great Ormond Street Hospital for Children, NHS Foundation Trust Research Histopathology Service, London, UK; 6 Department of Paediatric Respiratory Medicine, Royal Manchester Children’s Hospital, Manchester University NHS Foundation Trust, Manchester, UK; 7 The Willink Metabolic Unit, Manchester Centre for Genomic Medicine, Manchester University NHS Foundation Trust, Manchester, UK; 8 Paediatric ENT Department, Royal Manchester Children’s Hospital, Manchester University NHS Foundation Trust, Manchester Academic Health Science Centre, Manchester, UK

## Abstract

**Background:**

Mucopolysaccharidosis (MPS) type IVA is a rare lysosomal storage disorder caused by aberrations of the N-acetyl-galactosamine-6-sulfatase (GALNS) enzyme. MPS IVA is associated with a wide gamut of respiratory and airway disorders that manifest in a continuum of severity. In individuals exhibiting severe phenotypic expression, terminal stages of the disease frequently culminate in life-threatening, critical airway obstruction. These manifestations of end-stage disease are engendered by an insidious progression of multi-level airway pathologies, comprising of tracheomalacia, stenosis, tortuosity and 'buckling'. Historically, the management of end-stage airway disease has predominantly leaned towards palliative modalities. However, contemporary literature has posited that the potential benefits of tracheal resection with aortopexy, performed under cardiopulmonary bypass (CPB), may offer a promising therapeutic option. In this context, we report on outcomes from patients undergoing a novel approach to tracheal resection that is combined with manubrial resection, leading to improved airway calibre, obviating the requisition for CPB.

**Results:**

In this study, seven patients with severe MPS IVA exhibited clinical symptoms and radiological evidence indicative of advanced airway obstruction. All patients had a tracheal resection with a partial upper manubriectomy via transcervical approach, which did not require CPB. The surgical cohort consisted of 5 females and 2 males, the median age was 16 years (range 11-19) and the median height was 105.6cm (range 96.4-113.4). Postoperatively, significant improvements were seen in forced expiratory volume in 1 second (FEV1), with a mean increase of 0.68 litres (95% CI: 0.45-0.91; SD: 0.20). Notably, other spirometry variables also showed meaningful improvements, providing evidence of positive treatment effects. Furthermore, there were no major long-term complications, and the procedure resulted in a significant enhancement in patient-reported domains using PedsQL (version 4.0).

**Conclusions:**

This study represents the largest case series to date, on tracheal resection in patients with severe MPS IVA. Our findings demonstrate the effectiveness of the transcervical approach with partial manubriectomy for improving respiratory function and quality of life for individuals with advanced airway obstruction. Tracheal resection presents a promising treatment modality for severe cases of MPS IVA. Successful outcomes rely on meticulous multidisciplinary assessment, judicious decision-making, and appropriate timing of tracheal surgery. Further research and long-term follow-up studies are warranted to validate the long-term efficacy and safety of this approach.

**Supplementary Information:**

The online version contains supplementary material available at 10.1186/s13023-024-03253-3.

## Background

Mucopolysaccharidosis type IVA (MPS IVA; Morquio Syndrome [OMIM #253000]) is a rare, incurable, autosomal recessive lysosomal storage disorder (LSD) that is caused by a deficiency of the enzyme N-acetyl-galactosamine-6-sulfatase - GALNS [1, 2]. Whilst there is heterogeneity in severity and phenotype, patients with MPS IVA rarely survive past their second decade of life [1–3]. Most children with MPS IVA cease growing in height before the age of eight years, are wheelchair-bound by adolescence, and require multiple surgical interventions throughout their lifetime. The growth imbalance in MPS IVA is attributed to the differential effects of the enzyme deficiency and the resultant accumulation of glycosaminoglycans (GAGs) on various tissues and organs [4, 5]. Incomplete ossification, coupled with progressive dysplasia of the bones and cartilage, leads to characteristic skeletal abnormalities including short stature, a bell-shaped chest, spinal curvature, joint malformations, and an enlarged skull. While the skeletal growth decelerates and halts around age eight years, other parts of the body, such as the heart valves, cornea, trachea, and organs, continue to grow (or turn over) and accumulate keratan sulphate and chondroitin-6-sulfate [1, 4, 5]. This ongoing growth and GAG deposition contribute to complications such as cardiac disease, visual impairment, airway obstruction, and organomegaly. Notably, the skeletal pathologies, such as the gibbus deformity, frequently present as the initial clinical features of MPS IVA, and airway compromise remains the leading cause of mortality [1, 3, 4, 6, 7].

### Airway and respiratory disease in MPS IVA

**Fig. 1:**
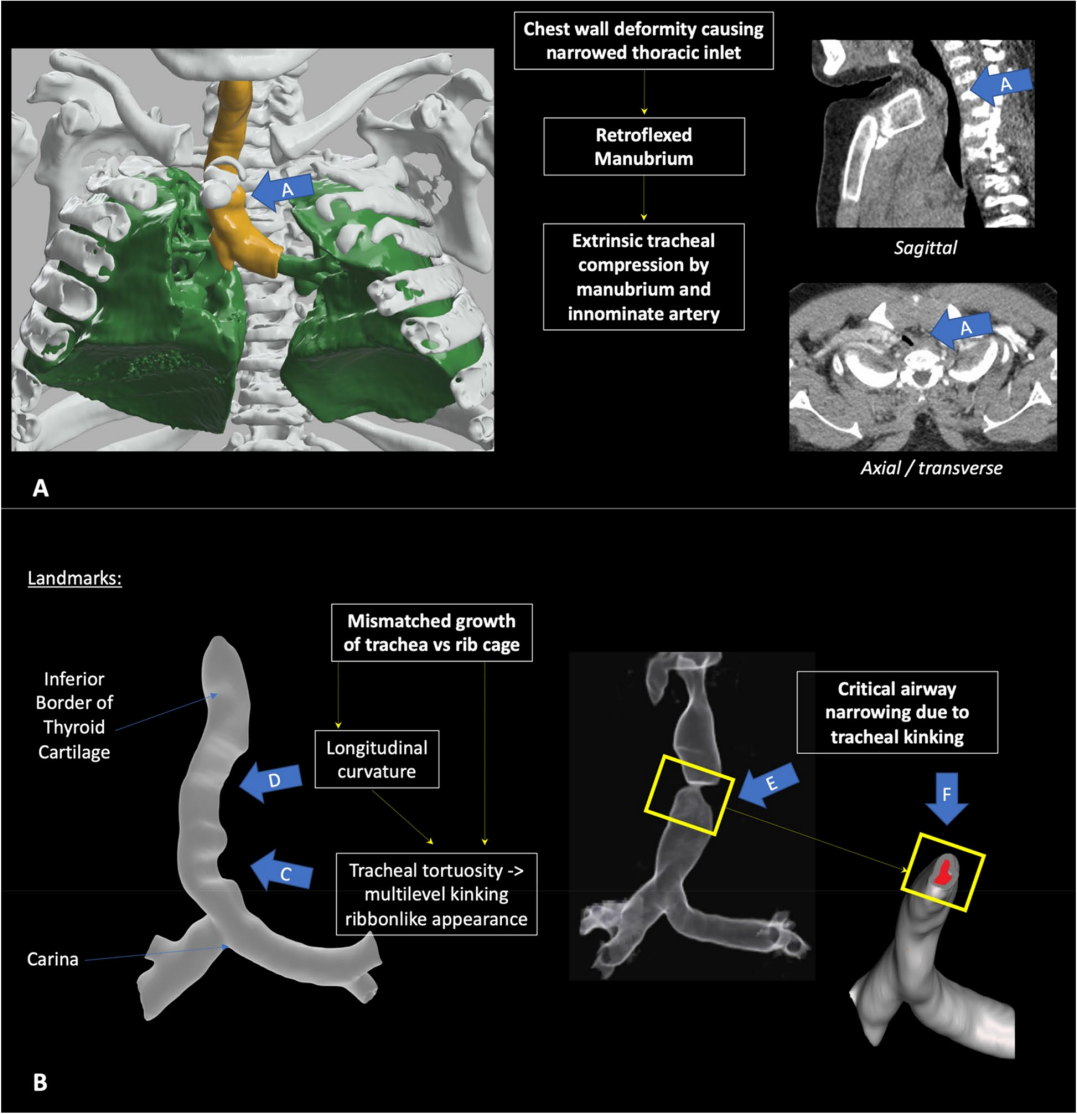
Airway Pathology in MPS IVA. Legend: Summary of the pathological processes causing airway obstruction in MPS IVA. **A**: 3-dimensional volumed rendered imaging (VRI) of trachea and thorax (left side), note contrast with CT imaging in sagittal and axial planes. Ribcage deformities such as pectus carinatum and narrowed thoracic inlet results in crowding of the intrathoracic structures (**A**; Blue arrow **A**). Abnormal manubrial retroflexion can displace the innominate artery and produce extrinsic compression of the trachea, at the thoracic inlet (Blue arrow **A**). **B**: 3D- tracheal reconstructions from CT for S3 and S4. The normal growth of the trachea, juxtaposed with the abnormal and restricted rib cage, causes multilevel kinking and the characteristic ribbon-like appearance (Arrow **C**). This growth mismatch also causes a longitudinal curvature (Arrow **D**). S4 had a single-level obstruction with relatively normal tracheal above and below the stenotic region (Arrow **E**), with an axial slice of the tracheal model illustrating the intraluminal area at the level of obstruction (Arrow **F**)

Airway and respiratory disease are ubiquitous in patients with MPS IVA, and despite significant advances in diagnostics and therapeutics, the condition continues to generate a substantial burden on patients, their carers, as well as providers of health and social care [1, 5, 8]. In severe phenotypic expression, progressive multi-level pathology develops within both the upper and lower respiratory tracts. This evolves into a composite interplay of obstructive airway and restrictive pulmonary diseases, ultimately leading to respiratory failure and premature mortality [1, 6, 9]. Pathological large airway disease includes tracheomalacia and tracheal tortuosity or ‘buckling’ of the trachea (see Fig. 1) [1, 3, 6, 10]. Tortuosity of the trachea is thought to reflect an imbalance between the relatively ‘normal’ longitudinal growth of the trachea and the abnormally short thoracic cage resulting from chest wall deformity and kyphoscoliosis [1]. Abnormal connective tissue metabolism has also been postulated as contributing to airway and respiratory disease through thoracolumbar kyphoscoliosis, reducing the diameter of the thoracic inlet and significant restrictive airway disease [1, 9, 11].

The introduction of enzyme replacement therapy (ERT, elosulfase alfa), multidisciplinary working, and centralised care has led to an increase in both lifespan and quality of life for individuals with MPS IVA [1, 4, 12]. However, ERT is not *curative* and does not stop progression or reverse skeletal dysplasia. Likewise, the airway can rapidly decompensate despite ERT and maximal supportive therapy, such as non-invasive ventilation (NIV) [1, 3, 9]. Pathologies are emerging as affected individuals live longer, and our understanding of the disease and efficacy of therapeutic interventions evolve. Clinicians are now challenged with patients living longer with better quality of life (QOL) and requiring new procedures which perhaps would have been historically inappropriate [13].

The MorCAP study by Harmatz et al. (2013) delineated that individuals with a severe phenotype of Morquio A syndrome typically ceased growth by eight years of age, with a final height below 120 centimetres being characteristic of this phenotype [5]. Tomatsu et al. (2016) evaluated 23 MPS IVA patients with obstructive central airway disease. Their study utilised computed tomography (CT) angiography of the chest to meticulously measure the tracheal cross-sectional area at various levels, providing critical insights into the extent and nature of airway obstruction within this patient population [6]. They ascertained that the degree of tracheal narrowing was inversely correlated with height, meaning that the shorter the patient, the more severe the tracheal narrowing. They also described three types of tracheal shapes in MPS IVA patients: normal, W-shaped, and T-shaped, with the W- and T-shaped tracheas were associated with focal tracheal narrowing and increased risk of airway obstruction [5]. These findings suggest that height can be used as a marker of disease severity and that tracheal shape can indicate the pattern of obstruction in MPS IVA patients.

While tracheal narrowing has long been recognised as contributing to progressive airway obstruction in MPS IVA it is only recently that there has been a concentrated emphasis on exploring surgical interventions as a means to alleviate tracheal stenosis. We had previously posited that the characteristic tortuosity of the trachea results from longitudinal loading of the trachea resulting from the mismatched growth between the shortened thoracic cage and relatively ‘normal’ length trachea. The initial tortuosity occurs at the level of the thoracic inlet due to the additional lateral pressure from the retroflexed manubrium associated with the gibbus deformity. Continued longitudinal pressure leads to further airway deformation at multiple levels, culminating in the pathognomic ‘ribbon-like’ appearance indicative of advanced stages of the disease [1].

### Surgical interventions for airway obstruction

Traditionally, palliative care represented the sole recourse for patients with severe airway complications such as near-fatal, critical airway obstruction. Non-invasive ventilation (NIV) is often employed in end-stage disease to maintain airway patency by splinting the upper airways open throughout the repository cycle, thereby mitigating dyspnoea. Whilst tracheostomy can be considered in isolated upper (oropharyngeal) airway obstruction, for lower tracheal obstruction and multilevel airway disease, tracheostomy may not ‘bypass’ the obstruction and in some cases, may not even be feasible. If a tracheostomy is inserted, acute airway obstruction can often ensue due to malpositioning of the tube itself in a deformed airway. Tracheal resection to ameliorate critical airway obstruction by resecting the stenosed segment(s) to improve respiratory mechanics has previously been explored. Pizarro et al. (2016), Kiessling et al. (2020), and Hack et al. (2020) detail this procedure conducted via a median sternotomy coupled with major vascular translocation necessitating CPB [14–16]. Their findings underscored significant enhancements in both patient-reported outcomes and overall quality of life.

Children currently being considered for airway surgery typically have stigmata of end-stage disease, such as near-fatal airway obstruction, thus presenting a very high anaesthetic risk. Furthermore, undertaking a median sternotomy, major vascular translocation and reimplantation of the innominate artery, and the requirement for cardiopulmonary bypass substantially increase perioperative risk [17, 18]. Furthermore, stenting of the stenosed tracheal segment has previously been considered but has seldom been undertaken successfully with demonstrable improvement in either QOL or long-term pulmonary function [9, 19].

This study expands upon our team's initial findings, where we previously detailed a case report employing the aforementioned surgical approach [20]. Herein, we chronicle our experience with seven adolescents who successfully underwent tracheal resection using an innovative transcervical method combined with partial manubriectomy. This strategy obviated the necessity for median sternotomy, cardiopulmonary bypass, and vessel translocation.

## Methods

### Aims

We aimed to delineate and better characterise end-stage, near-fatal tracheal obstruction in MPS IVA, introducing a safe and effectual novel surgical transcervical approach without the inherent risks of cardiopulmonary bypass. Additionally, we sought to explore available clinical outcomes and describe our multidisciplinary team (MDT) approach to surgical planning that included an independent ethical review for a novel, albeit potentially high-risk, procedure.

### Study design and patient selection

In this prospective case series, consecutive patients were evaluated through MDT assessment and pre-procedure planning. Of the eight patients assessed by the MDT, seven proceeded with surgery, while one was determined to be unsuitable for the procedure. Clinical data was prospectively collected from patient records that included demographics, clinical measurements (spirometry, oximetry, cardiorespiratory endurance: 6-min walk test - 6MWT), medical therapy (ERT infusions, concurrent medications) as well as biochemical (uKS, GAGs), radiological (computed tomography - CT, 3-dimensional reconstructions, virtual endoscopy) and QOL) metrics.

### Imaging

From multiplanar CT images, the percentage narrowing calculated using a methodology that improved upon the approach employed by Tomatsu et al. [6]. We firstly dividing the tracheal length into the equidistant segments, starting at the inferior border of the thyroid cartilage and ending at the carina. For each segment, the narrowest and widest calibre was noted. Thus for the stenotic region, we calculated the percentage narrowing for the surrounding segment, as this accounts for the topological variation that naturally occurs in the trachea, and unlike previous methodologies for scoring the tracheal narrowing, this would more likely account for multi-level disease.

We also have adopted an intricate methodology for assessing airway pathology, as detailed in Table 3. This method, which we have termed Advanced Airway Analytics (AAA), incorporates three-dimensional reconstructive modelling (3D-recons), 3D-printed models, and virtual endoscopy (VE). These tools empower the MDT with a virtual examination of the airway, assisting in the deliberation regarding prospective tracheal resection.

### Statistical analysis

We comprehensively analysed the treatment effects on spirometry outcomes in paediatric patients with end-stage airway disease who underwent tracheal resection. We employed both frequentist and Bayesian statistical approaches to evaluate the pre and post-operative spirometry data. Demographic data were summarised using means with standard deviations (SD) for continuous variables and medians with interquartile ranges (IQR) for skewed variables. The normality of spirometry data was assessed using Shapiro-Wilk tests, QQ plots, and histograms, confirming their approximate normal distribution and justifying the use of parametric tests.

Bayesian analysis via Markov Chain Monte Carlo (MCMC) simulations was explored to determine the posterior distributions for the mean differences in spirometry variables. The MCMC approach, with its 10,000 iterations, was chosen to effectively explore the full parameter space and ensure convergence to the true posterior distribution, thus providing reliable estimates. Our Bayesian model integrated prior information derived from previous spirometry results [11]. The resulting posterior analyses offered measures such as means, standard deviations, and 95% credible intervals, details of which can be found in Additional Data File 1. In our analytic framework, demographic data was integrated, normality was rigorously assessed, and both frequentist and Bayesian approaches were employed. This comprehensive strategy was devised to robustly assess the treatment effects on spirometry outcomes in patients undergoing tracheal resection for end-stage airway disease,

### Outcome measures

Our primary outcome measure focused on the safety and efficacy of the surgical procedure. We evaluated this by examining surgical and anaesthetic complications, the necessity for rescue extracorporeal heart-lung support, extended invasive ventilation on the intensive care unit, and the potential need for subsequent tracheal surgeries; spirometry metrics served as our physiological measure of surgical outcomes. Secondary outcomes encompassed changes in global quality of life (QOL), assessed using the PedsQL version 4.0 (Paediatric Quality of Life Inventory; Varni 2002) [21]. The PedsQL (v4) is a comprehensive and validated instrument tailored to measure changes in health-related quality of life in paediatric populations. The instrument comprises several domains, including physical functioning, emotional functioning, social functioning, and school functioning. Each domain provides insights into different facets of a child's well-being and the impact of medical interventions on their daily lives. We also monitored changes in the requirement for respiratory support.

Haematoxylin & Eosin (H&E) and immunohistochemistry to LAMP1 & CD68 was performed and optimised by the GOSH Research Histopathology service using clinically-employed antibodies (CD68(cloneKP1): Dako/CE-IVD CATMO814, Agilent USA; LAMP1: ab24170, abcam UK). Slides were digitally scanned using a high-resolution slide scanner (Aperio GT450 DX platform, Leica) and interpreted using QuPath software v0.4.3. Sizes of cartilage lacunae were quantified using H&E-stained slides by setting conservative pixel thresholds to the minimum levels that automatically captured all surrounding cartilage (confirmed by eye) and recording the % area of total cartilage that did not meet this threshold. LAMP1 and CD68 staining distribution was qualitatively analysed by eye.

All patients (as well as their parents when applicable) provided informed consent to publish clinical data in this case series, including the usage of imaging and histopathology. The manuscript follows PROCESS (2020) and SCARE (2021) reporting guidelines.

## Results

### Demographics and patient characteristics

Table 1 delineates the demographic and clinical characteristics of these patients, including age at tracheal resection, sex, ethnicity, presentation, GALANs mutation, consanguinity, age at diagnosis, and enzyme replacement therapy (ERT) treatment status. The patients had a median age of 17 years, ranging from 11 to 19 years. The genotypic profiles revealed a diversity of mutations, encompassing homozygous, heterozygous alterations, and in some instances, unidentified genetic markers. It is pertinent to mention that MPS IVA is frequently viewed as lacking a definitive genotype-phenotype correlation. Besides patients S7 (who had never been treated with ERT) and S6 (who ceased treatment due to a lack of demonstrable benefit), all patients were receiving weekly ERT infusions (at 2mg/kg dosage). Furthermore, each exhibited features of end-stage airway disease pathognomonic in MPS IVA, specifically critical (near-fatal) airway obstruction, causing profound functional limitations.

**Table 1:** Baseline characteristics

	**S1**	**S2**	**S3**	**S4**	**S5**	**S6**	**S7**	**S8**^**a**^
**Age at tracheal resection (years)**	17	13	16	19	17	16	15	11
**Sex**	Male	Female	Female	Female	Male	Female	Female	Male
**Ethnicity**	White, British	White, British	White, British	White, British	Arab	Asian, Pakistani	White, British	Asian, Pakistani
**Presentation**	Difficulty walking	Chest deformity	Difficulty walking / Scoliosis	Gibbus	Gibbus	Gibbus	Gibbus	Gibbus
**Gene mutation**	Homozygous p.(A291T)	Heterozygous p.(arg251Ter)	Homozygous p. (His166Arg)	Heterozygous p.(Gly155Arg)	No recognised gene	Homozygous c.347G>T (p.Gly116Val)	Homozygous p.l113F in exon 4 (p.ile113 phe, c.337A>T)	No recognised gene
**Consanguinity**	No	No	No	No	Unknown	Yes	No	No
**Age at diagnosis (months)**	70	43	30	12	42	10	36	22
**ERT Treated**	Yes	Yes	Yes	Yes	No	Stopped 2021	Yes	Yes
**Age at initiation of ERT (months)**	78	69	67	87	-	119	77	39
**ERT duration (years)**	10.9	8.8	11.9	12.2	-	9.9	6.5	9.5
**Height**^**b**^** (cm)**	109.0	105.9	103.4	107.0	102.0	106.0	96.4	113.4
**Weight **^**b**^** (Kg)**	29.4	19.5	26.1	34.9	37.0	26.0	18.0	38.3
***Neurological***	Previous craniocervical decompression and C-spine fixation	Previous craniocervical decompression and C-spine fixation	Cranio-cervical spinal stenosis with cervical cord myelomalacia	Previous craniocervical decompression and C-spine fixation	Cervical myelopathy (slight distal paresis)	Cervical myelopathy and decompression, Spinal fusion	Significant kyphotic (swan neck type) deformity	Craniocervical decompression and stabilisation
***Musculoskeletal***	Significant skeletal dysplasia Thoracolumbar kyphosis and bilateral hip dysplasia and arthrosis	Genu valgum (8-plates fixation)	Skeletal dysplasia Severe hip dysplasia with bilateral hip dislocation and coxarthrosis	Skeletal dysplasia Severe hip disease Recurrent ulna dislocation	Skeletal dysplasia Thoracolumbar kyphosis	Kyphoscoliosis Osteonecrosis both hips	Kyphoscoliosis	Skeletal dysplasia Genu valgum (8-plates fixation)
***Cardiac***	Nil	Mild mitral regurgitation	Mild-moderate aortic regurgitation	Nil	Nil	Cardio-myopathy mild tricuspid regurgitation	Cardio-myopathy mild tricuspid regurgitation and mitral stenosis	Mild mitral regurgitation
***Others***	Inguinal hernia (right-sided)	Bilateral sensorineural hearing defect	Dental caries Sensorineural hearing defect	Glanular hypospadias	Hearing (previous tympanoplasty)	Cauda equina Urinary incontinence	-	Dental caries

*ERT* Enzyme replacement therapy with weekly elosulfase alfa infusions, *PMH* Past medical history. All subjects were assessed to have severe airway disease and underwent multidisciplinary, multicentre evaluation for tracheal resection

^a^S9 was considered not a suitable candidate for surgery as airway pathology was comparatively less severe, and thus, the perceived risks from surgery outweighed potential benefits

^b^Height and weight measurements were recorded immediately prior to surgery

### Preoperative investigations

Preoperative findings derived from clinical investigations and examinations are delineated in Table 2. Spirometry findings in our study revealed a characteristic combination of restrictive and obstructive patterns commonly seen in severe MPS-IVA (Table 3). The specific changes observed in spirometry variables are discussed below.

**Table 2:** Summary of preoperative investigations

**Investigation**	**S1**	**S2**	**S3**	**S4**	**S5**	**S6**	**S7**	**S8**
**Respiratory**								
**6MWT (metres)**^**a**^	5	104	195	5	70	210	45	Unable to complete
**Spirometry**								
*-FEV1 (L/min)*	0.56	0.69	0.30	0.28	0.59	0.50	0.51	0.48
*-FEV1% pred*	37	52	23	20	42	43	48	40
*- FVC (L)*	1.19	1.07	0.48	0.58	0.67	0.79	0.61	0.63
*- FVC % pred*	73	76	36	40	45	64	54	48
*- FEV1/FVC:*	0.47	0.64	0.63	0.48	0.88	0.63	0.83	0.66
**Oximetry**	Severe SDB	Severe SDB	Mild SDB	Severe SDB	SDB	Mild SDB	Severe SDB	SDB
-mSpo2	94%	87.7%	95.2%	84%	93%	95%	92.9	94%
-ODI	17	62.3	3.7	27	16	2.5	24.9	11
-AHI	2.7	33.5	2.2	12	16	2.5	23.5	5.8
**NIV Treated**	BIPAP 16/8	CPAP 8	No	CPAP 10	No	No	BiPAP 8/4	CPAP 8-10
**Radiographic: Tracheal pathology from CT**								
**- Stenosis**	++	++	++	+++	+++	+++	++	+
**- Tortuosity**	+++	+++	+	-	++	+++	+++	+
**- Kinking**	++	+++	++	+	+++	++	+++	++
**- Multi-level disease**	+++	+	+++	-	++	++	+++	+
**- Thoracic inlet crowding**	++	+	+++	++	+++	+++	+++	+++
**- Retroflexed manubrium**	++	++	+	++	+++	++	++	++
**- Narrowest segment (mm)**	2.0	4.0	2.5	1.8	1.2	1.5	1.4	3.50
**- % Narrowing**	83	57.1	67.1	87.7	90.0	83.3	84.6	52.7
**Airway Assessment**								
**Laryngoscopy Grade (CL)**	1	2	1	2b	Unknown	2A	2	2
**Endotracheal Intubation Technique**	GlideScope	DL, Boogie	GlideScope DL	GlideScope DL	Unknown	GlideScope DL	DL	GlideScope
**Macroglossia**	No	No	Yes	Yes	No	Yes	No	Yes
**Clinical**								
**Previous adenotonsillectomy**	Yes	Yes	No	No	No	No	No	Yes
**MDT decision for Tracheal Resection**	Yes	Yes	Yes	Yes	Yes	Yes	Yes	No^b^

*6MWT* Six-minute walk test, *AHI* Apnoea-hypopnea index (episodes per hour), *CL* Cormack Lehane grading (1-4) at laryngoscopy, *DL* Direct laryngoscopy with rigid Hopkins and ETT mounted on, *FEV*_*1*_ Forced expiratory volume in one (first) second, *FEV*_*1*_*%pred* FEV_1_ as a percentage of predicted, *FVC* Forced vital capacity, *FVC %pred* FVC (actual) as a percentage of predicted for age, *mSpo2 %* median percentage oxygen saturations, *ODI 3%* Oxygen desaturations index, *SDB* Sleep-disordered breathing as diagnosed by polysomnography

^a^mobility assessed as part of managed access program prior to tracheal resection

^b^The multidisciplinary team (MDT) determined that the patient was not suitable for tracheal resection

**Table 3:** Postoperative findings, complications and outcome

	**S1**		**S2**		**S3**		**S4**		**S5**		**S6**		**S7**	
**Intraoperative and early postoperative**														
**Trachea resected (millimetres)**	16		30		30		30		35		30		35	
**Airway grading (DL) Cormack Lehane**	I		I		I		IIa		III		IIa		IIa	
**Aortopexy**	No		Yes		Yes		No		No		No		No	
**Need for Chest drain**	Yes, upper left post-operatively		Yes left sided		No		No		No		No		Yes, bilateral intra-operatively	
**Other Immediate complications**	No		No		No		No		Yes ^b^		No		No	
**Days until downsize**	7		3		7		7		3		3		3	
**ICU length of stay (days)**	9		4		7		7		5		5		5	
**Fit for discharge (days)**	14		6		10		8		10		8		7	
**Total length of inpatient stay (days)**	15		6		12		9		15		8		9	
**Postoperative spirometry (6 months)**														
	Pre	Post	Pre	Post	Pre	Post	Pre	Post	Pre	Post	Pre	Post	Pre	Post
**FEV1 (L/min)**	0.56	0.94	0.69	0.9	0.3	0.42	0.28	0.55	0.59	0.39	0.5	0.75	0.51	
**FEV1% pred**	37	63	52	67	23	32	20	40	42	28	43	65	48	
**FVC (L)**	1.19	1.31	1.07	1.09	0.48	0.55	0.58	0.61	0.67	0.44	0.79	0.79	0.61	
**FVC % pred**	73	82	76	76	36	41	40	42	45	30	0.64	0.64	54	
**FEV1/FVC:**	0.47	0.72	0.64	0.83	0.63	0.76	0.48	0.90	0.88	0.89	0.63	0.95	0.83	
**Complications and outcome after 6-12 month**														
**Late complications**	No		No		No		No		No		No		No^c^	
**NIV**^**a**^	Discontinued (16/8)		Discontinued (CPAP 10)		(No NIV previously)		BIPAP 16/4 (CPAP 10)		No		(No NIV previously)		Discontinued	

The table above describes the length of tracheal resected, the need for aortopexy, early and late postoperative complications, length of stay - paediatric intensive care unit (PICU) and total inpatient, and post-operative respiratory findings. The values of the spirometry tests were taken after six months post-procedure; we also note the values pre-surgery in parentheses. Note

^a^ - NIV – non-invasive ventilation includes continuous positive airway pressure – CPAP and bi-level positive airway pressure – BIPAP. The requisite need for NIV, the level off support post-surgery are stated (all indices are in cmH20), and values pre-surgery are in parenthesis.

^b^ - posterior glottic granulation tissue and stenosis above repair, secondary to the endotracheal tube

^c^ - Although a tracheal resection was performed within six months of publication, complete post-operative spirometry for this patient was unavailable at the time of writing. DL- direct laryngoscopy

The airway grading from previous laryngoscopies was noted using the Cormack-Lehane (CL) classification system (grades 1-4), in which grade 1 signifies a complete view of the glottis and relative ease of endotracheal intubation, and grade 4 denotes the inability to visualise the glottis or epiglottis structures [22]. Subject S3 was noted to be grade 1, S2 was grade 3, with the remaining patients being grade 2 CL; S5 had no prior information on airway grading. Exercise tolerance of the patients was evaluated using the 6-minute walk test (6MWT). This test has been utilised in clinical trials and is endorsed by NICE within a managed access agreement (MAA) framework, underpinning the reimbursement criteria for the therapy [23]. All patients exhibited significantly diminished 6MWT; however, the accuracy of this test as an evaluation of cardiorespiratory reserve may be confounded by the presence of severe musculoskeletal and neurological disability necessitating wheelchair utilisation. Additionally, all subjects exhibited varying degrees of sleep-disordered breathing (SDB), presenting symptoms of obstructive sleep apnoea and hypoventilation as evidenced by sleep studies. Subjects S1, S2, and S8 had previously undergone adenotonsillectomy. Subjects S1, S2, S4, S7, and S8 necessitated significant respiratory support from non-invasive ventilation (NIV) at night, without supplementary oxygen.

#### Advanced airway analytics and radiographic characteristics

Our analysis leveraged both conventional imaging (i.e CT) and Advanced Airway Analytics (AAA) to comprehensively evaluate airway pathology in the seven patients that underwent tracheal resection. Radiographic investigations, uncovered a spectrum of tracheal pathologies. These included multilevel and complex tracheal tortuosity, marked tracheal stenosis (at single or multiple levels, with or without deviation), with the narrowest tracheal segments ranging from 1.1 mm to 3.97 mm. Furthermore, pathological features included a retroflexed manubrium leading to significant thoracic inlet crowding and significant proximal tracheal kinking.

Quantitative assessment of the imaging identified a range of tracheal narrowing from 52.7% to 90.0% before surgical intervention, indicating the severity and variability of airway disease within our cohort (Table 2). Specifically, patients S1, S3, and S7 exhibited significant multi-level disease, presenting with the classic ribbon-like appearance indicative of advanced tracheal pathology. These patients demonstrated considerable tortuosity and stenosis, underscoring the complex nature of their airway obstruction. In contrast, patient S4’s pathology was characterised by relatively minor tortuosity, with a singular, significant area of stenosis, highlighting the heterogeneity in disease presentation among individuals with MPS IVA.

**Fig. 2:**
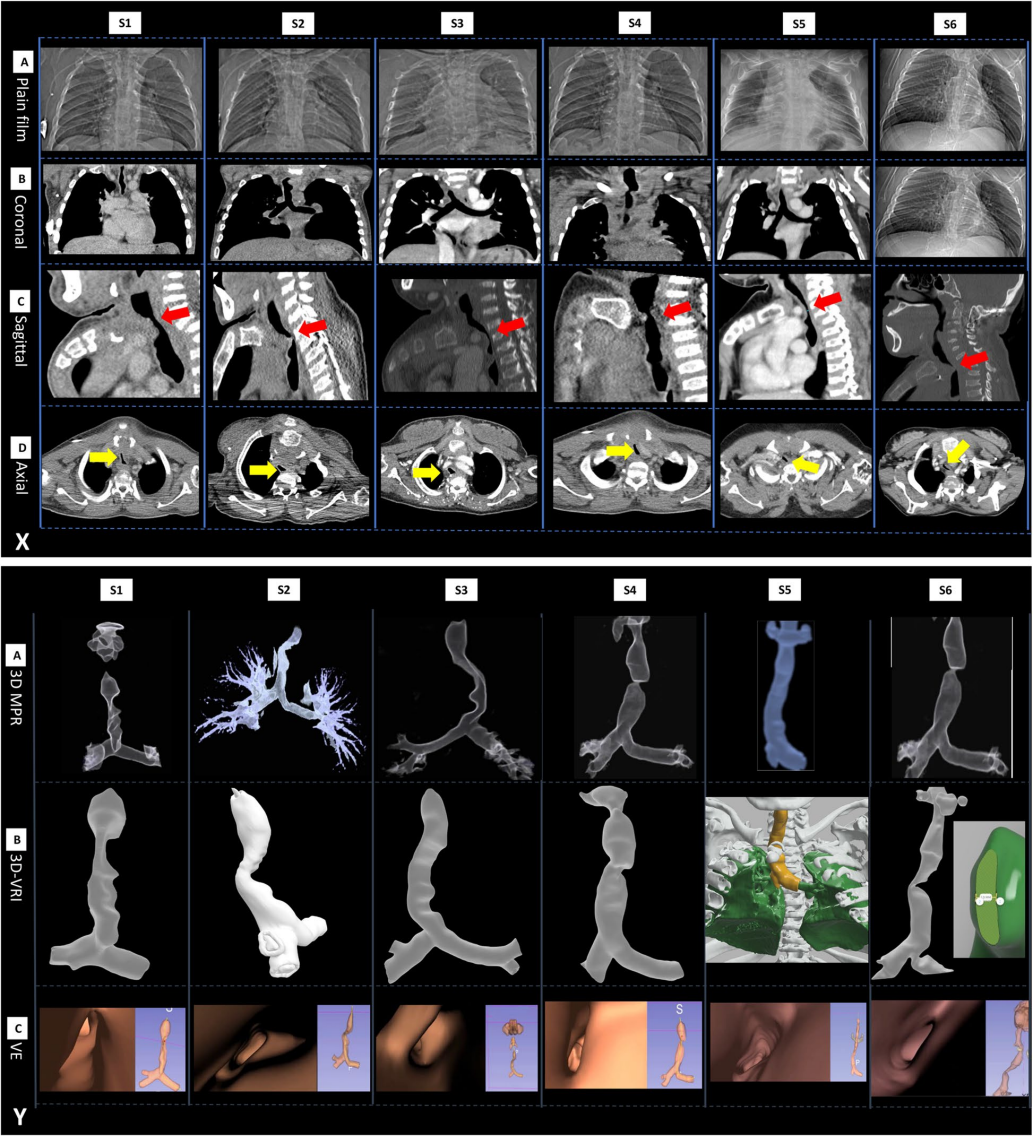
Contrasting 3D Reconstructed Tracheal Models to Conventional CT imaging. Legend: **X**: Conventional CT imaging of 9 patients. Views included a plain XR film (panel **A**), coronal (**B**), sagittal (**C**), and axial (**D**). Note the fully retroflexed manubrium that is orientated in a horizontal plane in all patients causing external compression of the tracheal at the level of the thoracic inlet (red arrows in the sagittal plane - **C** and yellow arrows in an axial slice **D**). **Y**: Advanced Airway Analytics (AAA) for patients (S1-6) illustrating 3D multiplanar (MPR) – **A**; 3D volume-rendered images (VRI) – **B**; virtual endoscopy (VE) segment – **C**. Note the clear superiority in visualisation and characterisation of tracheal anatomy and pathology for AAA pathology, when compared to conventional CT imaging (**X**)

The AAA approach, integrating three-dimensional reconstructive modelling, 3D-printed models, and virtual endoscopy, offered unparalleled insights into these structural challenges. This methodology was pivotal for the MDT’s surgical planning, facilitating a detailed virtual examination of the airway pathologies. Figure 2 compares conventional CT imaging to AAA, and Additional Data file 2 illustrates the virtual endoscopy undertaken preoperatively to facilitate multiplanar assessment of central airway pathology.

#### From referral to surgery: the MDT decision-making journey

Following extensive interdisciplinary discussions between the Royal Manchester Children's Hospital (RMCH) and Great Ormond Street Hospital (GOSH), the two largest and most experienced centres for treating children with inherited metabolic disorders in Europe. A robust consensus was reached to offer surgery to seven of the eight patients referred to MDT as an alternative to palliation. Ethical approval was obtained from the GOSH Ethics Committee. Several challenging factors required careful consideration, leading to a series of collaborative, interhospital multidisciplinary meetings with affected individuals and their families.

Eligible patients displayed severe pulmonary dysfunction from MPS IVA, unresponsive to ERT and non-invasive ventilation (NIV), significantly impairing their quality of life (QOL) and daily activities. Radiological evidence of tracheal pathology amenable to surgical intervention, such as stenosis, tortuosity, and severe tracheomalacia, was a prerequisite. On average, the timespan from referral to surgery was approximately 4-6 months. Notwithstanding, the criteria for surgery are intricately linked to the timing of intervention. We consider surgical options when patients display symptoms of end-stage central airway disease (near-fatal tracheal obstruction), alongside demonstrable inadequacy of conventional management strategies. Such inadequacy is substantiated by diagnostic evaluations including spirometry, polysomnography, or overnight oximetry. There is a delicate balance to identify the optimal time for surgery: the patient must be symptomatic enough to warrant the risks associated with surgery, yet not so critically ill that the perioperative risk becomes prohibitively high. In essence, surgery is contemplated when the benefits of addressing severe, life-threatening airway obstruction outweigh the potential risks of the procedure. Consent, which explicitly included the potential risk of death, was obtained for all cases. We direct readers to Additional Data file 1 for details on the referral pathway flowchart and the MDT referral documentation template.

### Perioperative considerations

#### Anaesthetic management

Anaesthesia was induced and maintained with total intravenous anaesthesia (TIVA) as a technique to avoid neuromuscular blockade and volatile anaesthesia that can impede and confound motor and sensory evoked potentials (MSEP). Moreover, TIVA is also beneficial in the context of surgeries on an open airway that could potentially result in the effumation of volatile anaesthetic gases and atmospheric pollution. Following induction of general anaesthesia, radial arterial cannulation, wide bore intravenous access was secured. Central venous cannulation was decided as not being merited since the procedure avoided ECLS. When the patients were at an adequate depth of anaesthesia, a laryngoscopy was performed, followed by a microlaryngospcopy (MLB) by the otolaryngologist and the airway was secured with an ETT. Given the need for prolonged neck extension with the incumbent risk of spinal cord compression throughout the procedure, spinal neuro-monitoring with MSEP/SSEP was performed at the start of the procedure, prior to airway instrumentation. This allowed for the delineation of baseline readings without neck extension, as well as ascertain thr maximal neck extension possible (to aid surgical access) without ensuing spinal cord compression, a process that was continuously monitored throughout the cases. Following a limited manubriectomy and reflection of the pretracheal tissue plane, exposing the trachea was transected, an ETT was sited into the distal trachea, with the anaesthetic circuit now being connected to the new ETT sited in the distal tracheal, in order to facilitate mechanical ventilation. The cases illustrate the prerequisite for excellent communication between the surgical, anaesthesia and neurophysiology teams, as there are often instances during the surgery where rapid fluctuation in the patient’s physiology warrants expeditious intervention.

#### Surgical technique

**Fig. 3:**
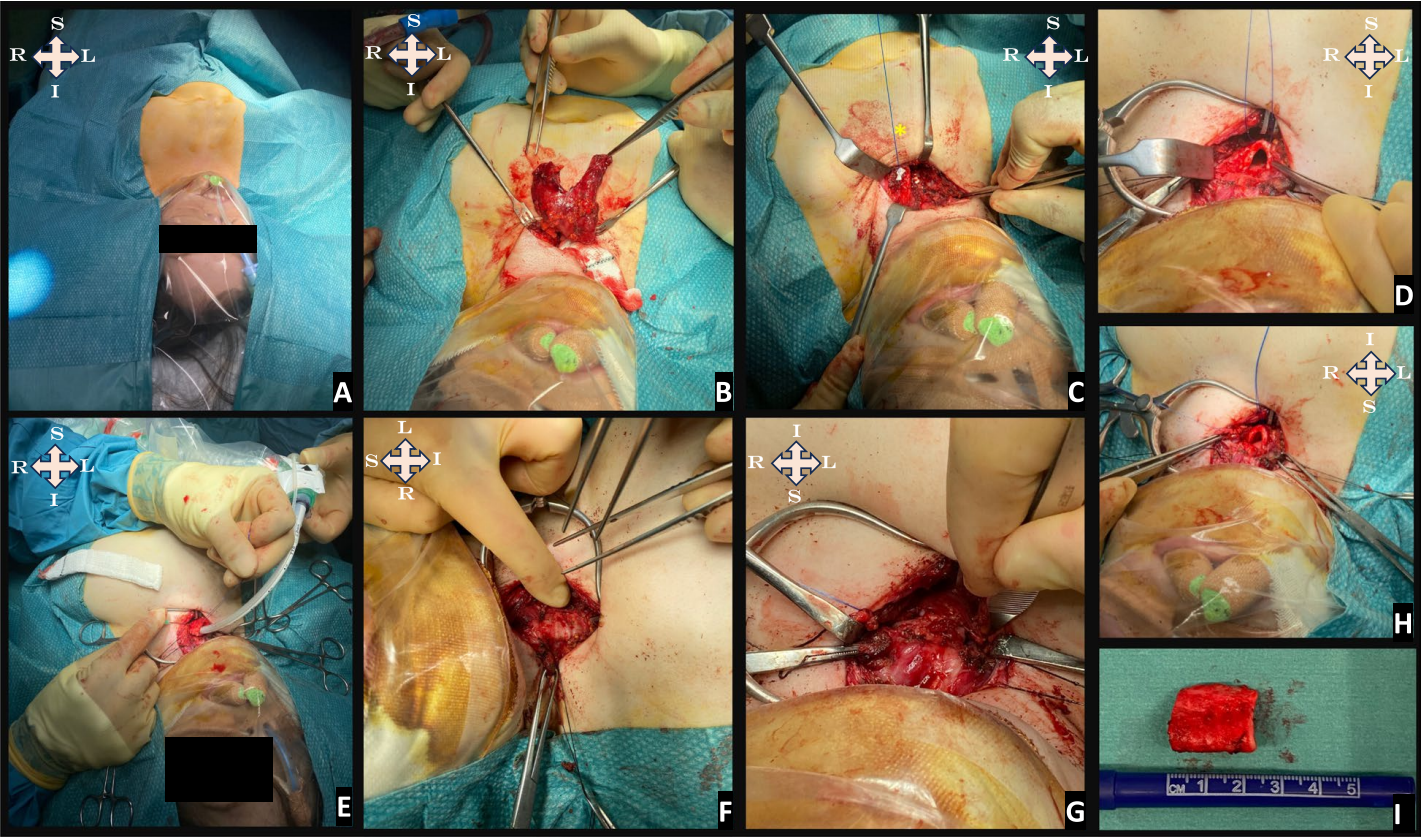
A Pictorial, Operative Journey of Tracheal Resection in Morquio Syndrome. Legend: This figure depicts the step-by-step process of tracheal resection in Morquio Syndrome: (**A**) illustrates the challenges of initial positioning due to a combination of cervical spine instability, kyphoscoliosis, and joint deformities; note the clear draping over the airway, a precaution taken to allow the anaesthesiologist to continuously monitor and maintain clear visibility of the airway. **B** Following exposure of the trachea an upper partial manubriectomy is performed. A thymectomy is typically performed to reduce anterior mediastinal crowding. **C** assessment of the deformed trachea is performed and sutures are placed for an aortopexy (*). **D** shows the exposure of the anterior trachea in the confines of the reduced sternomental distance. **E** depicts the tracheal transection, the cannulation of the distal trachea, and the extraction of the distal end over the proximal one to ascertain the redundant length of the trachea to be removed. **F** displays the omega-shaped morphology of the transected trachea and the optional placement of a temporary, intraoperative tracheostomy. **G** shows the initial anastomotic closure with lateral and posterior walls of the tracheal approximated, prior to the advancement of the nasotracheal tube over the repair line. **H** represents the completion of the tracheal anastomotic closure over the nasal endotracheal tube (ETT). **I** shows the resected tracheal specimen. To orient the reader, the anatomical positions are indicated as: S for superior, I for inferior, L for left, and R for right

For all patients, transcervical tracheal resection with upper partial manubriectomy was performed without cardiopulmonary bypass, as previously described by the authors [20]. A low midline cervical incision allowed for the dissection of the trachea, manubrium and clavicular heads. A partial manubriectomy was performed without disrupting the insertion of the clavicular heads. A thymectomy was often required giving further access to the upper mediastinum and the distal airway. The trachea was transected proximally below the level of the most proximal narrowing and dissected and mobilised off the oesophagus close the carina. In all children, the trachea was deformed in a concertina fashion, and following mobilisation and release by transection of the trachea would typically project beyond the cricoid. This would allow us to consider a minimum resection length in relation to the full length of the trachea. The median resection length was 30mm (*range*: 16-35mm; *interquartile range* -IQR: 9.5mm). We found that resecting a segmental length of around 30mm was optimal to achieve a tension-neutral anastomosis, in contrast to our initial experience (S1), where we were intentionally more conservative, resecting a segmental length of 16mm [20]. Primary end-on anastomosis of trachealis, the posterior trachea and its mucosa was performed, following which patients were re-intubated trans-nasally through the anastomosis under direct vision prior to closure of the anterior wall and temporary tracheostomy. Figure 3 provides a pictorial overview of the surgery, and Additional Data file 2 contains MLB video before and after the procedure.

#### Postoperative course

All patients were transferred to the paediatric intensive care unit (PICU) intubated and ventilated, allowing for a period of stability. Sedation was gradually weaned, with early multidisciplinary input that also encompassed physiotherapy and psychology, which improved and expedited immediate postoperative recovery. The patients were then extubated onto non-invasive ventilatory support (NIV), or nasal high-flow oxygen as a bridging therapy or simply oxygen via nasal cannula, as they embarked on their post-surgical recovery. Postoperative assessments revealed an absence of late complications. Temporary chest drainage was necessitated for patients S1, S2, and S7 in the perioperative phase. Rigorous neurological monitoring during the procedure safeguarded against lasting neurological consequences. Importantly, no instances of enduring nerve palsy were observed, and by three months, all patients had reverted to their preoperative functional baseline. Table 3 details postoperative findings, early complications, length of stay in the paediatric intensive care unit (PICU), and 6-12 month postoperative respiratory outcomes, including spirometry results and non-invasive ventilation (NIV) adjustments.

#### Histopathologic findings

**Fig. 4:**
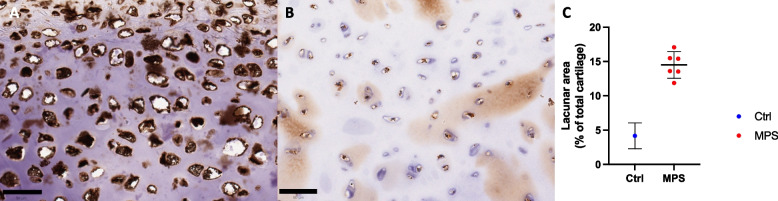
Characterisation of tracheal cartilage in patients with MPS IVA. Legend: Immunohistochemical staining for the lysosome marker LAMP1 showed florid staining within cartilage lacunae of MPS IVA children (**A**) in comparison to that seen within healthy control trachea (**B**). The lacunae themselves were also significantly enlarged in MPS IVA patients (**C**) (14.5% vs 2.4%, *p*=0.0035). Scale bar (50um)

Histological assessment included haematoxylin and eosin staining and immunohistochemistry for CD68 and LAMP1. Whilst no frank cartilage degradation or intracartilaginous macrophage infiltration was noted, chondrocyte lacunae were intrinsically significantly enlarged in MPS trachea compared to those seen in control paediatric tracheal cartilage (Fig. 4C, 14.5% of total cartilage area vs 4.2%, *p*=0.0035). LAMP1 staining intensity, typical of lysosomes engorged with storage material, was uniformly florid within these lacunae in comparison to normal trachea, regardless of ERT treatment status or duration.

### Changes in spirometry metrics

The analysis of the preoperative spirometry data revealed the following mean values: FEV1: 0.49 (95% CI: 0.35-0.62; SD: 0.15), FEV1% pred: 36.29 (95% CI: 25.95-46.62; SD: 11.18), FVC: 0.80 (95% CI: 0.56-1.03; SD: 0.26), FVC% pred: 53.14 (95% CI: 38.63-67.65; SD: 15.69), and FEV1/FVC: 0.63 (95% CI: 0.50-0.75; SD: 0.14). Postoperative spirometry (undertaken at 6 months post-procedure) exhibited notable improvements in FEV1, with a mean value of 0.68 (95% CI: 0.45-0.91; SD: 0.20). Furthermore, the remining spirometry variables such as FVC (forced expiratory volume in litres) also showed improvements, indicating positive treatment effects.

A significant positive effect on FEV1 following tracheal resection was confirmed by Bayesian analysis where the posterior mean difference was 0.800 (95% credible interval: 0.617 to 0.993). In contrast, the frequentist paired t-test did not yield a statistically significant result (*p* = 0.095). Similarly, FVC, showed improvement with mean difference of 0.572 (95% credible interval: 0.429 to 0.704. These findings highlight the clinical significance of the surgical intervention in improving respiratory function and support the use of both statistical approaches to comprehensively evaluate treatment effects (see Additional Data file 1 for Bayesian results). The clinical significance of these spirometry improvements is further highlighted by the correlation with patient-reported outcomes. Patients reported a significant reduction in breathlessness and a substantial improvement in breathing, aligning with the observed enhancements in spirometry metrics. These findings collectively underscore the positive impact of tracheal resection on respiratory function in paediatric patients with severe MPS IVA.

#### Postoperative microlaryngobronchoscopy

Microlaryngobronchoscopy was undertaken on postoperative day seven, which is aligned with our practice post-major tracheal resection as the National Tracheal Service for the United Kingdom. When compared to CT, MLB sanctions the early use of postoperative endoscopic examination. This confers a number of advantages, such as the direct scrutiny of the tracheal anastomosis, early detection (and degree) of intraluminal obstruction, dehiscence wound post-surgical granulations and enables therapeutic intervention such as the insertion of luminal stents.

#### Quality of life

The PedsQL (version 4.0) questionnaires, taken 6 months post-tracheal resection surgery, offer a clear insight into the marked improvements in quality of life. Additional Data file 1 contains a summary table of results from the PedsQL (v4.0) QoL questionnaire. Following tracheal resection surgery, responses was garnered from five out of the seven patients. In the self-assessments by the children, there was a conspicuous improvement in physical capabilities. Activities, such as walking beyond 100 metres, which previously were arduous, showed significant post-operative enhancement. The prevalent fatigue was now largely reported as mild to moderate. Emotional well-being also improved, with fewer instances of fear and sadness. Socially, fewer children felt isolated post-surgery. While academic and cognitive improvements were modest, a longer post-operative period might reveal more profound changes.

From the perspective of the parents, their observations aligned harmoniously with the self-reports from the children, underscoring the surgery's beneficial outcomes. Parents observed a discernible elevation in their child's physical proficiency. Emotionally, parents reported a well-balanced mental state for their child, with fewer occurrences of feelings of sadness or fear. In the context of social integration, the parent assessments also indicated a marked improvement post-surgery, suggesting a more seamless social transition for their child. On the academic front, parents echoed the improvements noted by their children, particularly emphasising reduced absenteeism from school or work due to health-related concerns. To summarise, the congruence between the child and parent reports substantiates the conclusion that tracheal resection significantly improves QOL. This accentuates the holistic benefits of tracheal resection, bolstering physical health, mental well-being, social engagment and overall quality of life.

## Discussions

Airway and respiratory complications are ubiquitous within the MPS spectrum and require a wide-reaching multidisciplinary approach to offer pragmatic and realistic therapeutic options in what remains an incurable disease. There is emerging evidence from long-term outcome studies that, despite treatment with ERT, airway disease and pulmonary function continue to deteriorate in many individuals [11]. ERT does not ameliorate the skeletal dysplasia, which compounds restrictive lung disease due to kyphoscoliosis and chest wall deformities [24]. We show here that this lack of treatment effect also appears to affect the hyaline cartilage of the tracheal rings, which are macroscopically more compliant at surgery and microscopically less dense. This creates a perfect storm combined of a trachea less inherently able to withstand compressive forces, thoracic inlet crowding from manubrial retroflexion and extrinsic compression from mediastinal neighbouring structures due to growth mismatch, and leads to an unrelenting decline towards profound tracheal deformity (i.e. stenosis, tortuosity and ‘buckling’) in severely affected patients. This is a key cause of morbidity and mortality in MPS IVA, as well as significantly increasing the risk of adverse perioperative outcomes during anaesthesia and surgery, even for simple surgical and radiological procedures [1, 9, 10]. Cervical spine disease (i.e. odontoid hypoplasia, atlantoaxial instability and cervical myelopathy) further compounds the risk due to the inherent chance of intraoperative spinal cord infarction, as well as creating technical difficulties in airway management in patients with cervical spinal fixation [4, 8, 15].

Until recently, the only therapeutic option for MPS IVA tracheal disease was palliation for symptom control, by either the institution or escalation of NIV. Tracheal stenting is seldom successful, with only a single reported case series exploring T-tube stents efficacy in three patients in which proximal granulation tissue formation was a significant complicating factor [19]. Intraluminal stents, whilst invaluable in the treatment of a variety of tracheal pathologies are often considered when there are no other surgical options. In MPS they are unable to overcome the significant compressive forces at play and often result in a chronic inflammatory response around the implant, resulting in the formation of granulation tissue and subsequent post-inflammation stenosis [17]. Moreover, MPS is associated with aberrations in multiple biological pathways, particularly lysosomal and cellular signalling, that can compound post-stenting granulation formation, causing rebound stenosis and accentuating the limitations of using tracheal stents in the MPS cohort of patients [25, 26].

In prior studies, Pizarro et al. (2016), Kiessling et al. (2020), and Hack et al. (2020) validated tracheal resection to ameliorate end-stage disease, employing a median sternotomy technique accompanied by extracorporeal cardiopulmonary bypass (CPB) [14–16]. The use of CPB and major artery translocation inherently augments the complexity and significantly increases the perioperative risks [18]. Collectively, these reports encompassed a total of four patients across three different international centres. We detail here a less invasive transcervical approach, with partial manubriectomy with or without limited aortopexy, which has provided targeted relief from extrinsic compression with symptomatic improvement whilst avoiding the major morbidity associated with CPB. The aforementioned case reports accessed the trachea through a median sternotomy, mandating extracorporeal life support (ECLS), either as traditional CPB with intrathoracic cannulation (Piazzo 2016 and Kiessling 2020), or extracorporeal membrane oxygenation - ECMO with central cannulation, Hack et al. (2020). Moreover, all modalities of ECLS exert a considerable impact on the perioperative risk. This impact manifests through a range of potential complications, such as haemorrhage, infection, embolic events, transfusion reactions, systemic anticoagulation, coagulopathy, arrhythmias, acute kidney injury, and aortic or major vessel dissection [18]. Notably, these risks are further exacerbated in an inherently very high-risk patient population, given the presence of severe, concomitant multisystemic comorbidities.

In guiding surgical interventions for end-stage MPS IVA, our study's MDT adhered to a comprehensive, ethically grounded decision-making framework. This approach meticulously balanced the severity of clinical manifestations against the associated perioperative risks. Ethical approval was obtained, reflective of the procedure's innovative yet high-risk nature. The patient selection process was anchored in a systematic evaluation of end-stage disease indicators, including critical tracheal obstruction and ineffectiveness of conventional therapies such as ERT and NIV. Consensus amongst the MDT was paramount in sanctioning surgical proceedings, ensuring that the intervention aligned with the patient's best interests. This approach was grounded in the principle that the therapeutic benefits of addressing severe airway obstruction decisively outweighing the surgical and anaesthetic risks. Patient and carer involvement in the decision-making process was pivotal, ensuring informed consent predicated on a thorough elucidation of the potential risks and merits. This patient-centric and inclusive approach to surgical candidacy and operative decision-making exemplifies the meticulousness and ethical rigour characterising our clinical methodology.

It is within this context of rigorous assessment and unanimous agreement that the case of patient S8 must be understood. S8, who exhibited symptoms of severe pulmonary dysfunction and was on maximal NIV, did not present with radiological evidence of critical obstruction that would render surgical intervention beneficial. Therefore, the MDT concluded that surgery would not adequately address the patient's pulmonary dysfunction and was not in the best interest of S8.

In conclusion, the surgical management of this cohort is encumbered by technical challenges and a nascent familiarity with the procedures, which is juxtaposed against the significant risk of anaesthetic adverse events, including notable difficulties in airway management characteristic of MPS IVA. Nonetheless, the experiences captured in our expanding case series, both in previous reports and the current study, have been both subjectively and objectively positive, demonstrating a remarkable improvement in patient-reported quality of life. It is now crucial to leverage these findings to iteratively refine the decision-making process, ensuring tracheal surgery is timed to maximise the benefit for each individual patient. Furthermore, the establishment of an international consensus on patient-centred outcome measures and composite outcomes would be invaluable, enabling a systematic evaluation of the prognostic benefits of therapeutic interventions within this rare disease cohort.

### Supplementary Information

Supplementary material 1.

Supplementary Material 2.

## Data Availability

The data that support the findings of this study are available from the corresponding author upon reasonable request. The datasets have been de-identified to ensure participant confidentiality. Researchers interested in accessing the data should contact the corresponding author.

## Abbreviations

3D-recons: Three-dimensional reconstructive modelling
6MWT: 6-minute walk test
AAA: Advanced Airway Analytics
CL: Cormack-Lehane classification system
CPB: Cardiopulmonary bypass
ECLS: Extracorporeal life support
ERT: Enzyme replacement therapy
ETT: Endotracheal tube
FEV1: Forced expiratory volume in 1 second
FVC: Forced vital capacity
IQR: Interquartile range
LAMP1: Lysosomal-associated membrane protein 1
MAA: Managed access agreement
MDT: Multidisciplinary team
MLB: Microlaryngobronchoscopy
MPS IVA: Mucopolysaccharidosis type IVA
MSEP: Motor and sensory evoked potentials
NICE: National Institute for Health and Care Excellence
NIV: Non-invasive ventilation
PICU: Paediatric intensive care unit
SDB: Sleep-disordered breathing
TIVA: Total intravenous anaesthesia
