# Supplementary material for: Novel approach for tracheal resection in Morquio a syndrome with end-stage critical airway obstruction: a UK case series
Source: Orphanet J Rare Dis. 2024 Jul 22;19:274. doi: 10.1186/s13023-024-03253-3 (PMC11264958; doi:10.1186/s13023-024-03253-3)

**Supplementary Information for: Novel Approach for Tracheal Resection in Morquio A Syndrome with End-stage Critical Airway Obstruction: A UK Case Series.**

Johnny Kenth FRCA, MSc^1,2^, Elizabeth Maughan MRCS(ENT) PhD^3,4^, Colin R Butler FRCS(ORL-HNS) PhD^3,4^, Jasleen Gabrie BSc^3^, Maral Rouhani^3^, Benjamin Silver^3^, Olumide K Ogunbiyi^5^**,** Stuart Wilkinson^6^, Reema Nandi FRCA, MD^3^, Robert Walker FRCA^1^, Nagarajan Muthialu FRCS^3,4^, Simon Jones MRCPCH^2,7^, *Richard Hewitt FRCS(ORL-HNS)^3^, *Iain A Bruce FRCS(ORL-HNS) MD ^2,8^

[1. MPS IVA: Tracheal Resection Referral Documentation (template) 2](#_Toc145607636)

[2. MPS IVA Tracheal Resection: Patient Referral Flowchart 4](#_Toc145607637)

[3. Qualitative Results from PedsQL (version 4.0) questionnaires 5](#_Toc145607638)

[4. Sample Vignettes: Post-Surgical Quality of Life Narratives from Patients and Parents 7](#_Toc145607639)

[5. Summary of Thematic Insights: Quality of Life Enhancements Following Surgery 8](#_Toc145607640)

[6. Bayesian Analysis Markov Chain Monte Carlo for ascertaining posterior distribution 10](#_Toc145607641)


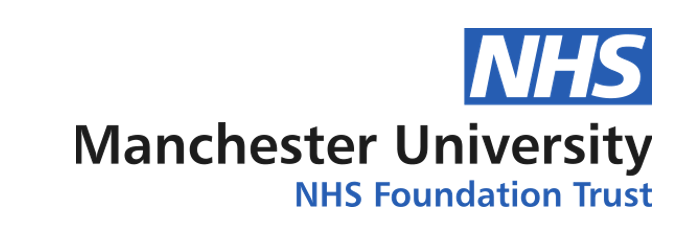

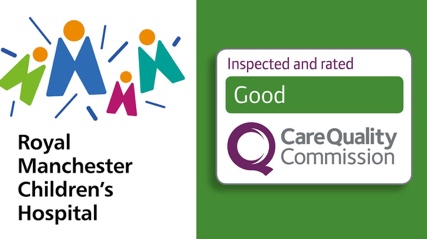

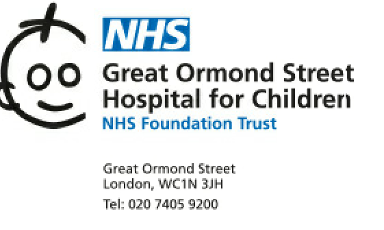


# MPS IVA: Tracheal Resection Referral Documentation (template)

| **Demographics** | UID (leave blank) |  | |
| --- | --- | --- | --- |
|  | **NHS number** |  | |
|  | **Name** |  | |
|  | **DOB** |  | |
|  | **Age (years)** |  | |
|  | **Gender** |  | |
|  | **Ethnicity** |  | |
|  | **Height (cms)** |  | |
|  | **Weight (kgs)** |  | |
| **Genetics & Disease Specific** | **Date of diagnosis (D/M/Y)** |  | |
|  | **Age at Diagnosis (D/M/Y)** |  | |
|  | **Presenting symptoms** at diagnosis |  | |
|  | **ERT** (Y/N) |  | |
|  | Date ERT started/ceased  (if applicable ) |  | |
|  | **Mutation(s) of GALNs  gene** |  | |
| **Respiratory History** | **Does the Child snore Or history of OSA (Y/N)** |  | |
|  | **Presence of severe airway disease** |  | |
|  | **Concomitant respiratory disease** |  | |
|  | **NIV** (date instituted, current settings, supplementary oxygen | |  |
| **Respiratory Investigations** | **Spirometry** | FEV1 (litres) |  |
|  |  | FEV1 [%predicted], |  |
|  |  | FVC (litres) |  |
|  |  | FVC [%predicted] |  |
|  |  | FEV1:FVC |  |
|  | **Polysomnography /**  **Overnight oximetr**y | Baseline arterial oxygen saturations (Spo2) |  |
|  |  | Mean nadir Spo2 |  |
|  |  | AHI [events/hour] -BTS |  |
|  |  | ODI 3% [events/hour] |  |
|  |  | ODI 4% [events/hour] |  |
|  | **6MWT** (6-min walk test) |  | |
|  | **3MSCT** (3-min stair climb test) |  | |
| **Airway & Respiratory Imaging** | **CT thorax** (findings + dates of previous scans) |  | |
|  | **Other thoracic imaging:**  MRI or CT Angio (if applicable) |  | |
|  | **Indirect or direct airway endoscopy**  (if applicable ) |  | |
|  | Latest Images transferred to RMCH + GOSH |  | |
| **Previous Anaesthesia History** | **Anaesthetic** **history**  Number of anaesthetic and any anaesthetic issues |  | |
|  | **Airway** grading  (Cormack-Lehane) |  | |
|  | **Other** peri-operative issues / complications |  | |
|  | Critical care admissions (including adverse incidents) |  | |
| **Previous Surgical History** | (*Previous operations - please list in reverse chronological order)* | | |
| **Other Past Medical History** | **CNS**  (i.e. C-spine fixation/decompression) |  | |
|  | **Cardiac** (structural / electrophysiological) |  | |
|  | **Musculoskeletal  & Orthopaedic** |  | |
|  | **Endocrine** |  | |
|  | **Others** |  | |
| **Other Investigations** | **MRI C-spine** |  | |
|  | **Cardiac**: Echo,  ECG |  | |
|  | **MAA data**  (please enclose previous 12 months) |  | |
| **Any further comments** |  | | |
| **Referral details** | **Referring centre** |  | |
|  | **Referring clinician** |  | |
|  | **IMD Consultant** if different from referring clinician |  | |

#
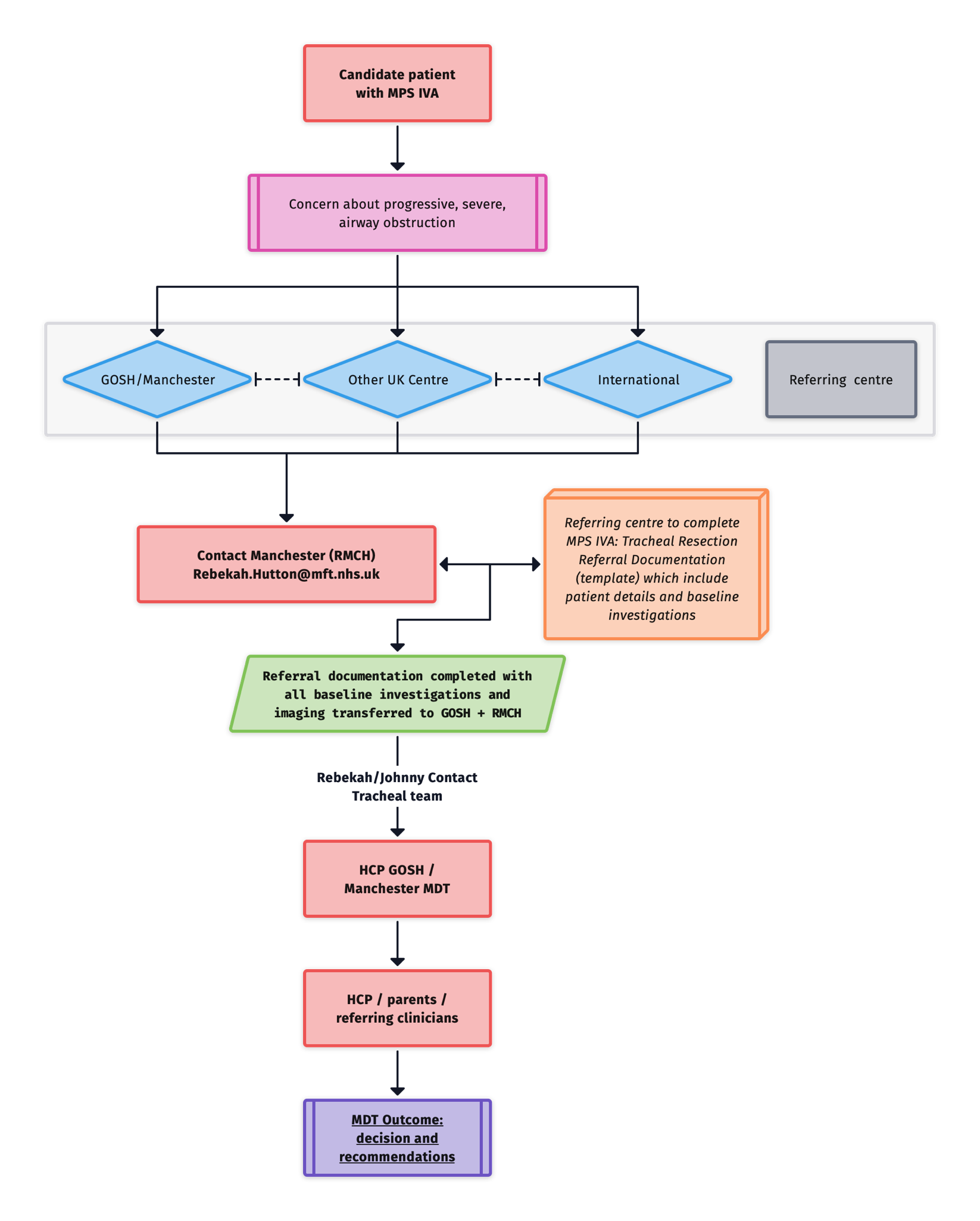
MPS IVA Tracheal Resection: Patient Referral Flowchart

# Qualitative Results from PedsQL (version 4.0) questionnaires

| C h I l d R e p o r t | Health and Activities | S1 | S2 | S3 | S4 | S6 | S7 |
| --- | --- | --- | --- | --- | --- | --- | --- |
|  | **1. It is hard for me to walk more than a couple of streets (~100 metres)** | 4 | 4 | 2 | 4 | 2 | 4 |
|  | **2. It is hard for me to run** | 4 | 1 | 0 | 4 | 1 | 3 |
|  | **3. It is hard for me to do sports activities or exercise** | 2 | 3 | 0 | 3 | 3 | 4 |
|  | **4. It is hard for me to lift heavy things** | 4 | 4 | 0 | 4 | 0 | 4 |
|  | **5. It is hard for me to have a bath or shower by myself** | 1 | 0 | 1 | 1 | 2 | 4 |
|  | **6. It is hard for me to do chores around the house** | 4 | 2 | 0 | 0 | 1 | 3 |
|  | **7. I have aches and pains** | 1 | 3 | 3 | 3 | 2 | 0 |
|  | **8. I feel tired** | 2 | 2 | 3 | 2 | 1 | 2 |
|  | **Feelings** |  | | | | |  |
|  | **1. I feel afraid or scared** | 0 | 3 | 2 | 1 | 2 | 0 |
|  | **2. I feel sad** | 0 | 0 | 3 | 1 | 1 | 0 |
|  | **3. I feel angry** | 0 | 3 | 3 | 0 | 1 | 1 |
|  | **4.I have trouble sleeping** | 2 | 1 | 2 | 1 | 2 | 0 |
|  | **5. I worry about what will happen to me** | 0 | 0 | 2 | 2 | 2 | 0 |
|  | **Get on with Others** |  | | | | |  |
|  | **1. I have trouble getting on with other children** | 0 | 0 | 2 | 4 | 0 | 3 |
|  | **2. Other children do not want to be my friend** | 0 | 0 | 1 | 0 | 1 | 2 |
|  | **3. Other children tease me** | 0 | 0 | 3 | 0 | 1 | 0 |
|  | **4. I cannot do things that other children my age can do** | 0 | 0 | 3 | 4 | 3 | 4 |
|  | **5. It is hard to keep up when I play with other children** | 0 | 0 | 3 | 2 | 3 | 4 |
|  | **School/Work** |  | | | | |  |
|  | **1.It is hard to pay attention at work/college/university** | 1 | 0 | 2 | 2 | 1 | 2 |
|  | **2.I forget things** | 1 | 2 | 3 | 2 | 1 | 3 |
|  | **3.I have trouble keeping up with my work or studies** | 2 | 0 | 3 | 1 | 1 | 2 |
|  | **4.I miss work or college/** **university because of not feeling well** | 0 | 0 | 3 | 2 | 2 | 4 |
|  | **5. I miss work or college/ university to go to the doctor or hospital** | 2 | 2 | 3 | 1 | 2 | 4 |
| P a r e n t R e p o r t | **Physical Functioning** | **S1** | **S2** | **S3** | **S4** | **S6** | **S7** |
|  | **1. Walking 100 meters** | 4 | 4 | 4 | 4 | 2 | 4 |
|  | **2. Running** | 4 | 4 | 4 | 4 | 3 | 4 |
|  | **3. Participating in Sports and activities or exercise** | 4 | 4 | 4 | 4 | 1 | 3 |
|  | **4. Lifting something heavy** | 3 | 3 | 4 | 4 | 4 | 4 |
|  | **5. Taking a bath or shower herself** | 3 | 3 | 4 | 2 | 2 | 3 |
|  | **Emotional Functioning** |  | | | | |  |
|  | **1. Feeling afraid or scared** | 0 | 1 | 2 | 2 | 2 | 2 |
|  | **2. Feeling sad** | 1 | 1 | 3 | 2 | 1 | 1 |
|  | **3. Feeling angry** | 0 | 1 | 3 | 0 | 1 | 2 |
|  | **4. Trouble sleeping** | 1 | 2 | 2 | 0 | 2 | 1 |
|  | **5. Worrying about what will happen to her** | 1 |  | 2 | 2 | 2 |  |
|  | **Social functioning** | 0 | | | | | 1 |
|  | **1. Getting on with other teenager** | 0 | 1 | 2 | 0 | 1 | 2 |
|  | **2. Other children not wanting to be her friend** | 0 | 0 | 2 | 0 | 1 | 2 |
|  | **3 Getting teased by other children** | 0 | 0 | 1 | 0 | 1 | 0 |
|  | **4. Not able to do things others her age can do** | 1 |  | 3 | 2 | 3 |  |
|  | **5. Keeping up with other young adults** | 1 |  | 3 | 2 | 3 |  |
|  | **School/Work Functioning** |  | | | | |  |
|  | **1. Paying attention at work or college/university** | 1 | 0 | 1 | 2 | 1 | 2 |
|  | **2. Forgetting things** | 1 | 1 | 1 | 2 | 1 | 3 |
|  | **3. Keeping up with work or studies** | 2 | 0 | 2 | 2 | 1 | 3 |
|  | **4. Missing work or college/university because of not feeling well** | 1 |  | 2 | 2 | 2 |  |
|  | **5. Missing work or college/university to go to the doctors/hospital** | 2 |  | 2 | 2 | 2 |  |

**Legend:** Results from the Quality of Life Questionnaire using PedsQL version 4.0 are displayed in the table above. Participants were asked to rate each item on a scale ranging from 0 to 4: 0 indicates *'Never a problem'*; 1, *'Almost never a problem'*; 2, *'Sometimes a problem'*; 3, *'Often a problem'*; and 4, *'Almost always a problem'*. Unanswered fields in the table are highlighted in dark blue.

# Sample Vignettes: Post-Surgical Quality of Life Narratives from Patients and Parents

|  | Patient Statement | Parent Statement |
| --- | --- | --- |
| S1 | *I’ve been able to do a lot more than I would have before the surgery. I don’t think I would have survived this past year mentally. I’ve just started university and wouldn’t have made it through without the surgery. I’ve finished seven weeks of 9-5 performances, felt more energetic post-surgery, reignited my passion for acting, am writing a book, my mood has improved, and I breathe more deeply with greater energy.* | *Prior to the surgery, he used most of his energy just to breathe. Physical and mental challenges were pronounced. Post-surgery, he breathed better, began physical rehabilitation, and started walking with assistance. The surgery has provided him a lot.* |
| S2 | *The surgery transformed my life. I no longer struggle with tasks like brushing teeth due to breathing issues. I breathe better during medical procedures, can hold my singing notes longer, and people around me noticed a huge difference in my breathing and walking post-surgery.* | *She is more energetic and mobile post-surgery. We saw a decline in her health before the surgery, but now her life has changed dramatically. She is more lively and even her school has noticed the improvement.* |
| S3 | *Breathing has improved post-surgery. I’ve begun reading books comfortably, continued swimming though with some challenges, and face some medical irritations. The surgery affected my underwater swimming.* | *The recovery was long due to throat swelling. However, she doesn’t get out of breath as easily now, and she can hold her head differently while breathing. Some surgeons are hesitant about further procedures. She felt positive post-recovery.* |
| S4 | *I’m not as breathless during daily activities now. Walking has become easier and I’m working on leg strength without getting as winded.* | *There’s a noticeable difference post-surgery. She switched from CPAP to BiPAP, indicating a change in her airway management. She enjoys reading and drawing more.* |
| S7 | *Post-surgery life has significantly improved. I engage in more hobbies, regularly participate in social activities like sleepovers, and feel more enthusiastic.* | *Everything has improved for her since the surgery. She sleeps better, functions better daily, and has a brighter outlook on life. Her physical appearance has improved, she’s happier, more energetic, enthusiastic about the future, and the surgery has dramatically changed our lives.* |

**Legend:** The table above presents vignettes capturing the perspectives of patients and their parents post-surgery. These narratives, expressed in their own words, shed light on the tangible and intangible improvements in the quality of life following the surgery, which tools such as PedsQL may fail to capture. Common thematic findings include enhanced physical abilities, markedly improved breathing, a boost in emotional and psychological well-being, positive external observations, swift recovery and adaptation, and increased social engagement.

# Summary of Thematic Insights: Quality of Life Enhancements Following Surgery


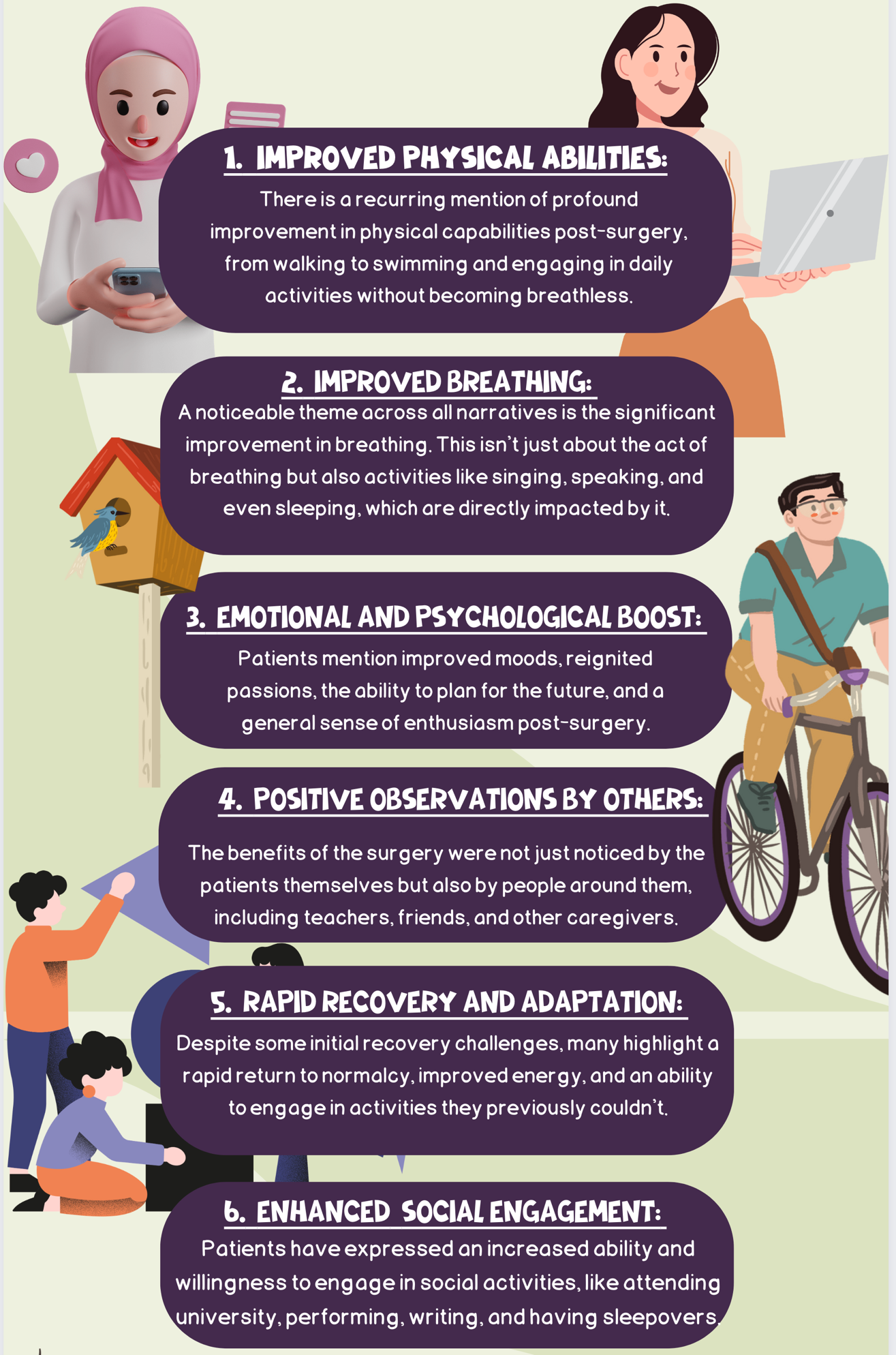
**Legend:**

The infographic above provides a graphical summary of the nuanced experiences of the patients and their caregivers post-surgery. To achieve this, we processed the results from the PedsQL (v4) questionnaire and patient vignettes through the advanced Artificial Intelligence (AI) capabilities of the GPT-4 Large Language Model (LLM). By leveraging Natural Language Processing (NLP) and complementing it with domain-specific fine-tuning and human-in-the-loop validation, we ensured the accuracy and relevancy of the extracted themes. The robustness and reliability of these AI-driven methods are corroborated by studies such as that by Gamieldien, Case, and Katz (2023), which delve into the potential of AI LLM and NLP in thematic coding.

1. *Gamieldien Y, Case JM, Katz A. Advancing Qualitative Analysis: An Exploration of the Potential of Generative AI and NLP in Thematic Coding. SSRN. June 21, 2023. Available from:*[*http://dx.doi.org/10.2139/ssrn.4487768*](https://dx.doi.org/10.2139/ssrn.4487768)

# Bayesian Analysis Markov Chain Monte Carlo for ascertaining posterior distribution

Summary of analysis of Bayesian analysis

Bayesian paired t-tests were conducted using Markov Chain Monte Carlo (MCMC) simulations with 10,000 iterations per chain. The posterior distributions of the mean difference for FEV1 and FVC were obtained from previous spirometry results, along with measures such as posterior means, standard deviations, and 95% credible intervals. The Bayesian analysis supported the findings from the frequentist tests. For FEV1, the posterior mean difference was 0.800 (95% credible interval: 0.617 to 0.993), indicating a positive effect of tracheal resection on the maximum amount of air forcefully exhaled in one second. Furthermore, the Bayes factor analysis provided evidence to reject the null hypothesis of no difference, with a Bayes factor of 3.373, supporting the presence of a significant difference in FEV1 before and after the procedure. In contrast, the frequentist paired t-test did not yield a statistically significant result (p = 0.095). Similarly, for FVC, the posterior mean difference was 0.572 (95% credible interval: 0.429 to 0.704), suggesting a significant improvement in the maximum volume of air forcibly exhaled after a full inhalation following the procedure.


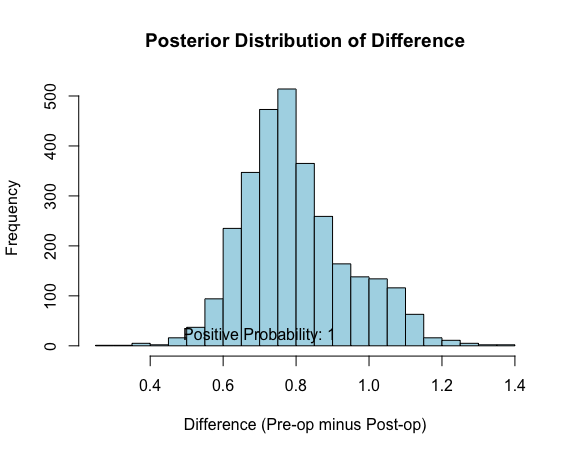


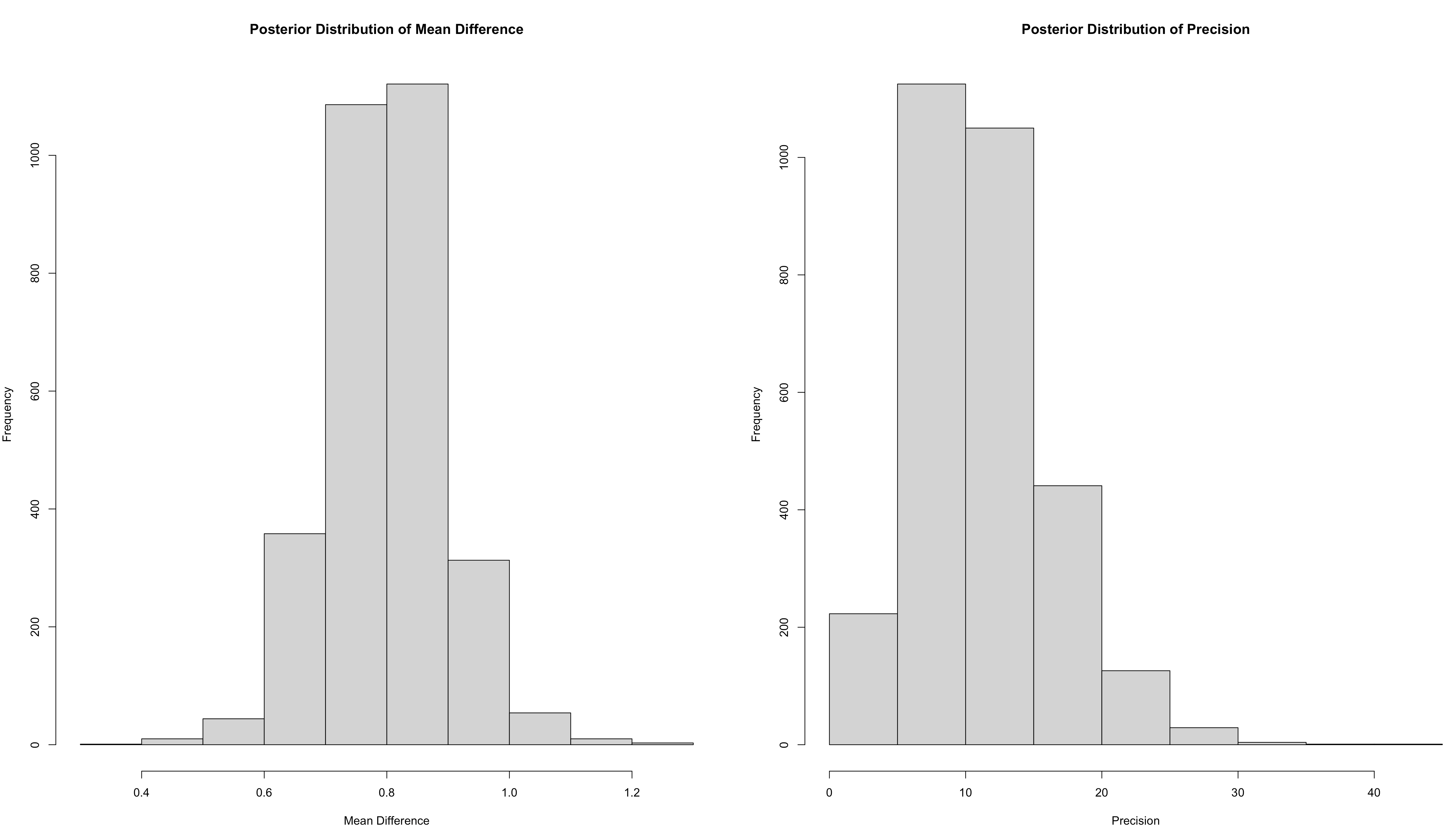

Supplement: Supplementary file 1 — Supplementary material 1. [file 13023_2024_3253_MOESM1_ESM.docx]
